# Clinical experience with glycerol phenylbutyrate in 20 patients with urea cycle disorders at a UK paediatric centre

**DOI:** 10.1002/jmd2.12386

**Published:** 2023-07-23

**Authors:** Mildrid Yeo, Preeya Rehsi, Megan Dorman, Stephanie Grunewald, Julien Baruteau, Anupam Chakrapani, Emma Footitt, Helen Prunty, Melanie McSweeney

**Affiliations:** ^1^ Department of Paediatric Inherited Metabolic Disease Great Ormond Street Hospital NHS Foundation Trust and Institute for Child Health London UK

**Keywords:** glycerol phenylbutyrate, hyperammonaemia, Ravicti, sodium benzoate, sodium phenylbutyrate, urea cycle disorders

## Abstract

In urea cycle disorders (UCDs) ammonia scavenger drugs, usually sodium‐based, have been the mainstay of treatment. Increasingly, glycerol phenylbutyrate (GPB, Ravicti®) is being used but scant real‐world data exist regarding clinical outcomes. A retrospective study of UCD patients initiated on or switched to GPB was performed at a UK centre. Data on population characteristics, treatment aspects, laboratory measurements, and clinical outcomes were collected before and after patients started GPB with a sub‐group analysis undertaken for patients with ≥12 months of data before and after starting GPB. UCDs included arginosuccinate synthetase deficiency (*n* = 8), arginosuccinate lyase deficiency (*n* = 6), ornithine carbamoyltransferase deficiency (*n* = 3), and carbamoyl phosphate synthetase 1 deficiency (*n* = 3). In the sub‐group analysis (*n* = 11), GPB resulted in lower plasma ammonia (31 vs. 41 μmol/L, *p* = 0.037), glutamine (670 vs. 838 μmol/L, *p* = 0.002), annualised hyperammonaemic episodes (0.2 vs. 1.9, *p* = 0.020), hospitalisations (0.5 vs. 2.2, *p* = 0.010), and hyperammonaemic episodes resulting in hospitalisation (0.2 vs. 1.6, *p* = 0.035) reflecting changes seen in the whole group. Overall, patients exposed to sodium and propylene glycol levels above UK daily limits reduced by 78% and 83% respectively. Mean levels of branched chain amino acids, haemoglobin, and white cell count were unchanged. Two adverse drug reactions (pancytopenia, fatigue/appetite loss) resolved without GPB discontinuation. Patients/families preferred GPB for its lower volume, greater palatability and easier administration. GPB appeared to improve biochemical measures and clinical outcomes. The causes are multi‐factorial and are likely to include prolonged action of GPB and its good tolerability, even at higher doses, facilitating tighter control of ammonia.

**Synopsis**

Patients who switched to glycerol phenylbutyrate from sodium‐based scavengers experienced improved ammonia control and fewer hyperammonaemic episodes and hospitalisations.

## 1 INTRODUCTION

The therapeutic goal when managing patients with urea cycle disorders (UCDs) is normal growth and development. In addition to a protein‐restricted diet, sodium‐based nitrogen scavengers (sodium benzoate, NaBz, and/or sodium phenylbutyrate, NaPBA) have been a mainstay of treatment to reduce the risk of hyperammonaemia and its sequelae. Despite these measures, clinical outcomes have remained variable. This may be related to a lack of adherence to these scavengers because of their poor palatability, large volume, and undesirable gastric effects. There are also challenges with maintaining therapeutic levels of sodium‐based scavengers because of their relatively short duration of action.^1^, ^2^, ^3^ A glycerol‐based scavenger, Ravicti® (1.1 g/mL glycerol phenylbutyrate, GPB) became available in the United Kingdom in 2018. It was designed with glycerol as its carrier to overcome the drawbacks of the sodium‐based scavengers. GPB is a low‐volume, tasteless, odourless liquid, that has been demonstrated to be more palatable than other sodium‐based scavengers, which supports adherence. It is free from sugar, sodium, and propylene glycol (PG). It is a triglyceride, not a salt, which has been shown to result in a slower speed of absorption, longer duration of action, and improved ammonia control compared to NaPBA.^4^ Clinical evidence supporting GPB as a well‐tolerated and effective medicine for UCD patients is growing.^5^, ^6^ Since GPB became available, increasing numbers of patients of various ages and UCDs have been transitioned to it from sodium‐based scavengers, and newly‐presenting patients have been initiated on it once they are metabolically stable. We present a retrospective analysis of clinical and biochemical data in patients receiving GPB to evaluate how GPB is affecting clinical outcomes.

## 2 METHODS

### 2.1 Study design

This was a retrospective observational study of UCD patients receiving treatment with GPB and managed according to standard clinical practice at a single centre in the United Kingdom (Great Ormond Street Hospital, London). Study data were obtained from electronic medical records and were collected where available for a period of up to 3 years before a patient started GPB and for the duration of treatment thereafter until July 2022. Ethical approval was not required but informed consent was obtained from every patient or their care giver as appropriate.

### 2.2 Study variables

Data from four categories were collected: population characteristics, treatment aspects, laboratory measurements, and clinical outcomes. Population characteristics included (i) sex, (ii) diagnosis, and (iii) age at initiation of GPB. Treatment aspects included (i) dose of scavenger replaced by or supplemented by GPB, (ii) starting dose of GPB, (iii) final titrated dose of GPB, (iv) sodium exposure, and (v) PG exposure. Laboratory measurements included (i) ammonia, (ii) glutamine, (iii) branched chain amino acids, (iv) liver function, and (v) blood count. Clinical outcomes included (i) protein tolerance, (ii) hyperammonaemic episodes (plasma ammonia ≥80 μmol/L) and hospitalisations, (iii) adverse events, and (iv) patient satisfaction.

Blood samples for laboratory measurements were taken at routine hospital appointments and in the outpatient setting, and if patients presented acutely unwell to hospital. Fasting state when blood samples were taken was not recorded. Plasma ammonia was measured by reflectance absorbance spectrophotometry at 600 nm after reaction with a specific indicator (bromophenol blue) using the VITROS® 5600 Integrated System (Ortho Clinical Diagnostics). Plasma amino acids were measured by absorption spectrometry at 570 nm of the ninhydrin derivative following protein precipitation with 5‐sulphosalicylic acid containing a specified quantity of internal standard (S‐(2‐aminoethyl)‐l‐cysteine hydrochloride [AEC]) and separation by cation exchange chromatography using the Biochrom 30+ Amino Acid Analyser Physiological System (Biochrom Limited).

### 2.3 Initiation of GPB

Patients were initiated on GPB to allow discontinuation of one or more intravenous or oral sodium‐based scavengers. The method of initiation of GPB depended on whether the scavenger being replaced was oral NaBz, oral NaPBA, or intravenous NaPBA. One patient was not receiving a sodium‐based scavenger and was initiated on GPB as add‐on therapy to carglumic acid (CGA). Once patients had been transitioned to GPB and their ammonia levels were confirmed to be under control, doses were titrated at a routine clinic appointment. The final titrated daily dose of GPB was based on the patient's protein requirements with consideration to their previous sodium‐based scavenger requirements.

#### 2.3.1 Switching from oral NaBz

The initial daily dose of GPB was that recommended by the manufacturer for phenyl‐butyrate‐naïve patients: 8.5 mL/m^2^/day (9.4 g/m^2^/day) in patients with a body surface area (BSA) < 1.3 m^2^, or 7 mL/m^2^/day (8 g/m^2^/day) in patients with a BSA ≥ 1.3 m^2^ (Ravicti® Summary of Product Characteristics [SPC], available at medicines.org.uk). Our approach evolved as our clinical experience grew. The initial approach for the first three patients involved a gradual phased transition over 1 or 2 weeks as described in Yeo et al.^7^ Subsequently, we practiced direct replacement, stopping NaBz and introducing GPB on the same day.

#### 2.3.2 Switching from oral NaPBA

The initial daily dose of GPB was as described in the SPC for Ravicti®, with patients receiving a dose of GPB containing the same amount of phenylbutyric acid (PBA) as the oral NaPBA it was replacing.

#### 2.3.3 Switching from intravenous NaPBA

Once stable with controlled ammonia, patients were transitioned from NaPBA (500 mg/kg/day) to GPB (12.5 mL/m^2^/day) over 2 days. GPB was introduced at a dose of 5.6 mL/m^2^/day, the dose of NaPBA was reduced to 250 mg/kg/day, then the dose of GPB was increased to 12.5 mL/m^2^/day and NaPBA discontinued.

#### 2.3.4 Initiating GPB as add‐on therapy to CGA

The initial daily dose of GPB was that recommended in the SPC for Ravicti® for phenyl‐butyrate‐naïve patients.

### 2.4 Statistical analysis

Statistical analysis was undertaken using the Microsoft Excel Analysis ToolPak. Population characteristics, treatment aspects, and clinical outcomes for all patients were analysed using descriptive statistics. Laboratory measurements were analysed by longitudinal comparisons for the periods before and after a patient started GPB. Where the initial acute or sub‐acute presentation was managed with intravenous scavengers, data from that episode of hyperammonaemia was not included in our dataset; data were collected once patients were stabilised and receiving oral scavenger treatment. Comparisons were undertaken by a paired *t*‐test for normally distributed variables and a Wilcoxon test for data that were not normally distributed. Where measurements of ammonia were below the limit of detection (<9 μmol/L) a pragmatic approach was taken, and a measurement of 8 μmol/L used as a substitute for the actual unknown value. A *p*‐value of <0.05 was regarded as statistically significant.

The study design meant that the amount of data for each patient before or after starting GPB varied, which meant longitudinal comparisons were not balanced. To provide a more balanced comparison, a sub‐group analysis was undertaken for laboratory measurements and clinical outcomes in patients with at least 12 months of data either side of starting GPB.

A second sub‐group analysis was undertaken for infants and neonates (3 months of age and below) which represent a group of interest regarding the efficacy of treatment with GPB. Longitudinal comparisons were undertaken for laboratory measurements for this sub‐group.

### 2.5 Annualised rates of hyperammonaemic episodes and hospitalisations

Due to the periodic nature of hyperammonaemic episodes and hospitalisations, annualised rates could only be reliably compared on an individual basis in the patients with at least 12 months of data either side of starting GPB. Other reported measures of annual rates before and after starting GPB in a particular group were calculated by summing instances in all patients in that group and dividing by the cumulative number of treatment years to provide an overall group mean.

## 3 RESULTS

### 3.1 Population characteristics

Twenty patients with UCDs were identified from 17 families (Table 1). The male to female ratio was 3:1. Four UCDs were represented with arginosuccinate synthetase deficiency (ASSD) (*n* = 8) being the commonest followed by arginosuccinate lyase deficiency (ASLD) (*n* = 6), ornithine carbamoyltransferase deficiency (OTC) (*n* = 3), and carbamoyl phosphate synthetase 1 deficiency (CPS1) (*n* = 3). Five patients (5/20, 25%) received prospective management from birth because they had a previously diagnosed sibling. Twelve patients (12/20, 60%) had acute presentations with hyperammonaemia at a median age of 4 days (range 0–8 days). Three patients (3/20, 15%) presented at age 1, 2, and 5 years respectively with vomiting and hyperammonaemia. All patients began treatment with GPB between January 2019 and June 2022. The median age for commencing GPB was 4 years (range 9 days to 15 years). The most common treatment change observed was from oral NaBz monotherapy to GBP (*n* = 11). The total duration of follow‐up for GPB was 40 years and 11 months.

**TABLE 1:** Population characteristics.

	Patients (*n* = 20)
Sex, *n* (%)	
Male	15 (75%)
Female	5 (25%)
UCD diagnosis, *n* (%)	
Ornithine carbamoyltransferase deficiency (OTC)	3 (15%)
Arginosuccinate lyase deficiency (ASLD)	6 (30%)
Arginosuccinate synthetase deficiency (ASSD)	8 (40%)
Carbamoyl phosphate synthetase 1 deficiency (CPS1)	3 (15%)
UCD onset, *n* (%)	
Prospective management at birth because of affected sibling	5 (25%)
Acute, within first 8 days of life	12 (60%)
Sub‐acute, within first 5 years of life	3 (15%)
Age at initiation of GPB in years, mean (SD)	5.0 (4.78)
Age group when initiated on GPB, *n* (%)	
<1 month	2 (10%)
1 month to 3 months	4 (20%)
4 months to 5 years	6 (30%)
6–11 years	6 (30%)
12–17 years	2 (10%)
Treatment change, *n* (%)	
NaBz to GPB	11 (55%)
NaPBA to GPB	2 (10%)
NaBz + NaPBA to GPB	3 (15%)
NaBz + NaPBA to NaBz + GPB	3 (15%)
CGA to CGA + GPB	1 (5%)
Scavenger(s) replaced by GPB, *n* (%)	
NaBz	11 (55%)
NaPBA	5 (25%)
NaBz + NaPBA	3 (15%)
Duration of data collection in months, mean (SD)	
Before starting GPB	22 (15.6)
After starting GPB	25 (13.9)

Abbreviations: CGA, carglumic acid; GPB, glycerol phenylbutyrate; NaBz, sodium benzoate; NaPBA, sodium phenylbutyrate; UCD, urea cycle disorder.

### 3.2 Treatment aspects

#### 3.2.1 Scavenger doses

Doses of NaBz and NaPBA replaced by GPB ranged from 91 to 500 mg/kg/day and 225 to 500 mg/kg/day respectively, with median doses of 300 and 245 mg/kg/day. The dose of CGA supplemented by GPB was 200 mg/kg/day.

The starting dose of GPB ranged from 4.8 to 12.5 mL/m^2^/day, with a median of 8.5 mL/m^2^/day. The final titrated dose of GPB ranged from 4.4 mL/m^2^/day (177 mg/kg/day) to 11.7 mL/m^2^/day (743 mg/kg/day), with a median of 8.8 mL/m^2^/day (436 mg/kg/day).

#### 3.2.2 Sodium exposure

Before starting GPB, 19/20 (95%) patients were receiving one or more sodium‐based scavengers; the mean daily sodium exposure was 1.4 g (SD 1.5 g) with 9/19 (47%) of patients being exposed to sodium levels above their recommended National Health Service (NHS) daily limits which have been derived from World Health Organisation (WHO) guidance (Table 2).^8^, ^9^ After starting GPB, three patients (3/19, 16%) remained on a sodium‐based scavenger (NaBz): a 6‐year‐old, a 14‐year‐old, and a 1‐year‐old had daily sodium exposures of 0.8 g, 4.2 g, and 0.9 g respectively, equivalent to 63%, 175%, and 109% of their daily recommended limits. The number of patients exposed to sodium levels above the recommended limits reduced by seven (7/9, 78%) after GBP was initiated.

**TABLE 2:** Treatment aspects.

	Before starting GPB	After starting GPB
Number of patients receiving each scavenger treatment		
NaBz, *n* (%)	17 (85%)	3 (15%)
NaPBA, *n* (%)	8 (40%)	0 (0%)
CGA, *n* (%)	1 (5%)	1 (5%)
GPB, *n* (%)	0 (0%)	20 (100%)
Total number of different treatments, *n*	26	24
Number of patients on multiple scavengers, *n* (%)	6 (30%)	4 (20%)
Treatment doses, mean (SD)		
NaBz, mg/kg/day	277 (101)	289 (151)
NaPBA, mg/kg/day	335 (137)	n.a.
CGA, mg/kg/day	200 (n.a.)	200 (n.a.)
Starting dose of GPB, mL/m^2^/day	n.a.	8.3 (1.98)
Titrated dose of GPB, mL/m^2^/day	n.a.	8.7 (2.18)
Sodium exposure above recommended limits, *n* (%)^a^		
<12 months (daily limit of 0.4 g sodium) (*n* = 6)	1 (17%)	0 (0%)
1–3 years (daily limit of 0.8 g sodium) (*n* = 4)	3 (75%)	1 (25%)
4–6 years (daily limit of 1.2 g sodium) (*n* = 4)	2 (50%)	0 (0%)
7–10 years (daily limit of 2 g sodium) (*n* = 3)	1 (33%)	0 (0%)
11 years and over (daily limit of 2.4 g sodium) (*n* = 3)	2 (67%)	1 (33%)
All ages (*n* = 19)	9 (47%)	2 (11%)
Propylene glycol exposure above recommended limits, *n* (%)^b^		
<28 days (daily limit of 1 mg/kg) (*n* = 1)	1 (100%)	0 (0%)
1 month to 4 years (daily limit of 50 mg/kg) (*n* = 7)	5 (71%)	1 (14%)
5 to 17 years (500 mg/kg) (*n* = 9)	0 (0%)	0 (0%)
All ages (*n* = 17)	6 (35%)	1 (6%)

Abbreviations: CGA, carglumic acid; GPB, glycerol phenylbutyrate; n.a., not applicable; NaBz, sodium benzoate; NaPBA, sodium phenylbutyrate.

^a^ National Health Service (NHS) guidance regarding sodium intake is derived from World Health Organisation (WHO) guidelines and is a maximum of 0.4 g in children <12 months, 0.8 g in children aged 1–3 years, 1.2 g in children aged 4–6 years, 2 g in children aged 7–10 years, and 2.4 g in children aged 11 years and over.^8^, ^9^

^b^ Royal College of Paediatrics and Child Health (RCPCH) guidance regarding propylene glycol intake is a maximum of 1 mg/kg/day in children under 28 days, 50 mg/kg/day in children aged 1 month to 4 years, and 500 mg/kg/day in children 5–17 years.^10^

#### 3.2.3 Propylene glycol (PG) exposure

Seventeen patients were receiving NaBz. The mean PG exposure from NaBz before starting GPB in neonates up to 28 days of age (*n* = 1), in children 1 month to 4 years of age (*n* = 7), and in those more than 4 years of age (*n* = 9) was 36 mg/kg, 57 mg/kg, and 46 mg/kg respectively. After starting GPB, 3 of the 17 patients (18%) remained on NaBz. A 6‐year‐old, a 14‐year‐old, and a 1‐year‐old had daily PG exposures of 37 mg/kg, 36 mg/kg, and 83 mg/kg corresponding to 7%, 7%, and 167% respectively of their maximum daily doses as advised by the Royal College of Paediatrics and Child Health (RCPCH).^10^ The number of patients exposed to PG levels above the recommended limits reduced by five (5/6, 83%) after GBP was initiated.

### 3.3 Laboratory measurements

Whilst the 20 patients were receiving treatment with GPB, the mean measurement of ammonia was lower (32 vs. 44 μmol/L, *p* = 0.006), of platelets was lower (333 vs. 370 × 10^9^/L, *p* = 0.035), and of gamma‐glutamyl transferase (GGT) was higher (19 vs. 16 U/L, *p* = 0.031) compared with the period before starting GPB (Table 3). There was a trend towards a reduction in glutamine, but it was not statistically significant (*p* = 0.057). There were no significant reductions in the mean levels of branched chain amino acids, haemoglobin, and white cell count.

**TABLE 3:** Laboratory measurements and clinical outcomes in the whole group and sub‐groups.

	Number of patients	Before starting GPB	After starting GPB	*p* value
**All patients, all data**				
Plasma ammonia, glutamine, and branched chain amino acids, μmol/L, mean (SD)				
Ammonia	20	44 (21)	32 (16)	0.006**
Glutamine	17	955 (788)	712 (272)	0.057
Leucine	17	68 (32)	61 (22)	0.353
Isoleucine	17	36 (15)	35 (13)	0.670
Valine	17	113 (36)	114 (27)	0.890
Liver function, mean (SD)				
ALT	17	55 (33)	48 (39)	0.378
AST	1	28 (n.a.)	15 (n.a.)	n.a.
GGT	6	16 (3)	19 (2)	0.031*
Full blood count, mean (SD)				
Haemoglobin, g/L	17	116 (13)	116 (16)	0.927
Platelets, ×10^9^/L	17	370 (105)	333 (81)	0.035*
White cell count, ×10^9^/L	17	11 (4)	11 (7)	0.120
**Patients with at least 12 months of data either side of starting GPB**				
Plasma ammonia, glutamine, and branched chain amino acids, μmol/L, mean (SD)				
Ammonia	11	41 (20)	31 (16)	0.037*
Glutamine	11	838 (212)	670 (135)	0.002**
Leucine	11	60 (20)	58 (13)	0.762
Isoleucine	11	34 (13)	34 (9)	0.970
Valine	11	112 (33)	116 (20)	0.710
Mean liver function, U/L (SD)				
ALT	11	51 (38)	37 (25)	0.083
AST	1	28 (n.a.)	15 (n.a.)	1.000
GGT	5	15 (2)	19 (2)	0.063
Mean full blood count, (SD)				
Haemoglobin g/L	11	123 (7)	124 (6)	0.645
Platelets ×10^9^/L	10	380 (90)	337 (73)	0.017*
White cell count ×10^9^/L	10	10 (5)	9 (5)	0.131
Clinical episodes per year, mean (SD)				
Hyperammonaemic episodes	11	1.9 (2.5)	0.2 (0.2)	0.020*
Hospitalisations	11	2.2 (2.0)	0.5 (0.7)	0.010**
Hyperammonaemic episodes resulting in hospitalisation	11	1.6 (2.2)	0.2 (0.2)	0.035*
**Neonates (less than 1 month) and infants (1–3 months)**				
Plasma ammonia, glutamine, and branched chain amino acids, μmol/L, mean (SD)				
Ammonia	6	37 (24)	30 (11)	0.350
Glutamine	4	1314 (1704)	677 (200)	0.875
Leucine	4	94 (52)	76 (40)	0.321
Isoleucine	4	43 (23)	44 (20)	0.906
Valine	4	126 (53)	122 (42)	0.848
Liver function, mean (SD)				
ALT	4	62 (25)	72 (60)	0.777
AST	0	n.a.	n.a.	n.a.
GGT	0	n.a.	n.a.	n.a.
Full blood count, mean (SD)				
Haemoglobin, g/L	3	91 (2)	93 (24)	0.750
Platelets, ×10^9^/L	3	431 (174)	376 (119)	0.750
White cell count, ×10^9^/L	3	15 (6)	15 (7)	0.750

*Note*: The data shown represent data for patients with measurements before and after starting GPB.

Abbreviations: ALT, alanine aminotransferase; AST, aspartate aminotransferase; GGT, gamma‐glutamyl transferase; GPB, glycerol phenylbutyrate; n.a., not applicable.

* Significant at the 5% level.

** Significant at the 1% level.

### 3.4 Clinical outcomes

#### 3.4.1 Protein tolerance

Dietary protein tolerance was unchanged throughout the course of the study. Dietary protein allowance was adjusted in line with usual practice, with target protein intake based on WHO recommendations.^11^

#### 3.4.2 Hyperammonaemic episodes and hospitalisations

Before introducing GPB there were 33 hyperammonaemic episodes in the 20 patients over a period of 14 treatment‐years, equivalent to an overall annualised rate of 2.3. After introducing GPB, there were 24 instances over 41 treatment‐years, representing a 74% reduction in the annualised rate of hyperammonaemia to 0.6. There was a 70% reduction in the rate of hyperammonaemic episodes resulting in hospitalisation (27 instances to 23 instances, equivalent to annualised rates of 1.9 and 0.6). We also observed a 49% reduction in hospitalisations for any cause (37 instances to 54 instances, equivalent to annualised rates of 2.6 and 1.3).

#### 3.4.3 Adverse events

There were no reports of adverse events except in two patients. One patient developed unexplained pancytopenia 16 months after starting GPB; the dose of GPB was left unchanged and symptoms resolved completely. One patient had an episode of fatigue and reduced appetite 28 months after starting GPB with incremental dose of GPB; their dose of GPB was reduced from 12.4 to 7.3 mL/m^2^/day and NaBz was introduced, then 10 months later the dose of GPB was increased to 9.9 mL/m^2^/day and the dose of NaBz was reduced, after which lethargy and appetite concerns resolved. It is uncertain whether the aetiologies of these adverse events were related to GPB.

#### 3.4.4 Patient satisfaction

Informal feedback from patients/family, recorded in the medical notes, demonstrated a preference for GPB for reasons such as its lower volume, greater palatability and easier administration.

### 3.5 Sub‐group analysis: patients with at least 12 months of data either side of starting GPB

Eleven patients had at least 12 months of data either side of starting GPB. The results of this sub‐group analysis largely reflected the findings in all 20 patients. Mean ammonia level was significantly reduced after starting GPB (41 vs. 31 μmol/L, *p* = 0.037). Mean glutamine, which had approached statistical significance in whole group analysis (*p* = 0.057) was now statistically significantly reduced (838 vs. 670 μmol/L, *p* = 0.002). Branched chain amino acids remained unchanged. GGT, with paired measurements available in just five patients, was increased but not statistically significant (15 vs. 19 U/L, *p* = 0.063) and remained within the normal reference range. Mean platelet count reduced significantly (380 vs. 337 × 10^9^/L, *p* = 0.017).

Before introducing GPB there were 21 instances of hyperammonaemic episodes in this sub‐group over a period of 11 treatment years (an overall annualised rate of 1.9). After introducing GPB, there were five instances over 32 treatment years, representing a 92% reduction in the annualised rate of hyperammonaemia to 0.2 (*p* = 0.020). Similarly, there was a 78% reduction in hospitalisations (24 instances to 15 instances, equivalent to annualised rates of 2.2 and 0.5) (*p* = 0.010), and a 90% reduction in the rate of hyperammonaemic episodes resulting in hospitalisation (17 instances to 5 instances, equivalent to annualised rates of 1.6 and 0.2) (*p* = 0.035) (Table 3).

### 3.6 Sub‐group analysis: neonates (less than 1 month) and infants (1–3 months)

Of the six neonates and infants, five (two neonates and three infants) were swapped directly from sodium‐based scavengers (three from NaBz, two from NaPBA) and one infant was started on GPB as an add‐on to treatment with CGA. A paired comparison of laboratory measurements before and after starting GPB was undertaken for patients, however, due to the small number of patients, no statistically significant differences were found (Table 2). During the cumulative 65 months of follow‐up after starting GPB, there were 16 hyperammonaemic episodes of which 15 resulted in hospitalisation. There were an additional 21 hospital admissions unrelated to hyperammonaemia. The annualised rates for hyperammonaemic episodes (3.0), hospitalisations (6.7), and hyperammonaemic episodes resulting in hospitalisation (2.8) were greater than the rates for all 20 patients and for that of the sub‐group of 11 patients with at least 12 months of data either side of starting GPB.

## 4 DISCUSSION

For years, sodium‐based scavengers have been a mainstay of treatment in UCD patients to reduce the risk of hyperammonaemia and its sequelae. However, these treatments have shortcomings. GPB is an alternative scavenger therapy and evidence from clinical trials and real‐world data studies suggest GPB is achieving better clinical outcomes than the earlier‐generation sodium‐based scavengers.^1^, ^5^, ^6^, ^7^, ^12^, ^13^, ^14^

In our study, the mean ammonia level decreased significantly overall once patients started GPB. Glutamine was also reduced significantly in the balanced sub‐group analysis. This is likely explained by the mechanism of action of GPB, which sees it converted to phenylacetate (PAA) in the body, which reacts with glutamine to form phenylacetylglutamine (PAGN), which is excreted in the urine eliminating excess nitrogen as a result. Additional reasons for the observed effects may relate to characteristics of GPB such as its ability to confer a steadier level of scavenger in the plasma compared with sodium‐based alternatives and that GPB is thought to be able to dispose more nitrogen than NaBz and NaPBA.^3^, ^4^ In addition, the relatively low volume and better palatability of GPB means that patients are able to take their full prescribed dose.^1^, ^2^

No significant changes were detected in branched‐chain amino acids. Liver function tests revealed no statistically significant differences after starting treatment with GPB other than in GGT. GGT levels remained within the normal range but increased in the few patients for whom we had measurements. This could be explained by expected age‐related increases.^15^

Blood count was assessed in our cohort due to a single patient who had unexplained pancytopenia whilst on GPB. Overall, there were no significant changes in blood counts except for a statistically significant decrease in platelets. Reasons for this observation could be expected age‐related changes and the reduction in sodium exposure upon commencing GPB, as high chronic sodium exposure has been associated with elevated platelet levels.^16^, ^17^ Regardless of the cause of the change in platelet levels the levels remained within the normal range, therefore this finding was not regarded as clinically significant.

As well as differences in ammonia control, there were differences in exposure to excipients. With sodium‐based scavengers, there is inevitable exposure to sodium additional to that already present in the diet. Introducing GPB allowed us to reduce the number of patients exposed to sodium above their daily recommended limits by 78%. Whilst sodium‐based scavengers are necessary in the acute phase of hyperammonaemia, consideration should be given to the sodium load they pose to patients who receive them as maintenance therapy. Chronic exposure to high levels of sodium has been shown to be strongly associated with hypertension and all‐cause mortality, incident cardiovascular disease, and non‐fatal coronary heart disease. Reduction of chronic sodium exposure could potentially prevent these unwanted sequelae.

Besides sodium, another excipient to consider is PG present in NaBz. This has been shown to accumulate and cause toxic effects when administered over a prolonged period. In November 2020, the RCPCH developed guidance on the daily exposure limits of PG in paediatric patients.^10^ This was prompted because exposure to PG was found to cause CNS depression, especially in neonates and young children, associated with hyperosmolality, metabolic acidosis, and renal impairment. Before starting GPB, daily PG limits were exceeded in six patients receiving NaBz with the greatest exposure in a neonate equivalent to 3600% of the recommended daily limit. After starting GPB, three patients remained on NaBz and only one patient exceeded their maximum daily dose. Those at greatest risk of PG overexposure are neonates and younger children and therefore are a group that require additional consideration when deciding on scavenger therapy.

The hospitalisation rates in neonates and infants were relatively high compared with the rest of the group. This could be due to challenges in achieving metabolic stability in the immediate period after diagnosis and because families would usually have a lower threshold to attend hospital with clinical concerns. Looking at the group as a whole, the longitudinal nature of our study means that statistically significant treatment related changes in biochemical and clinical parameters might be suggested to be a function of age‐related changes in metabolic stability. After all, metabolic stability can improve in patients as they grow older, although age‐related changes in glutamine, ammonia, and metabolic stability are not uniform and depend on multiple factors. For the group with 12 months of data either side of starting GPB, the median age at which patients started GPB was 7 years (range 2–15 years) and there were no apparent age‐related trends before the introduction of GPB that would explain the changes in ammonia levels and episodes of hyperammonaemia seen after the introduction of GPB. Our clinical interpretation is that the commencement of GPB, more than age, contributed to the improvements in metabolic stability of our patients. This translated into improved clinical outcomes with significant reductions in annualised hospitalisation rates.

Use of GPB allowed three patients to reduce from dual scavenger treatment to monotherapy. Being able to reduce the number of scavengers whilst maintaining metabolic stability has its obvious advantages and reduces the burden on both the patient and the health service.

Whilst the data presented reflects over 40 patient‐years of experience with GPB and therefore represents one of the largest real‐world studies to date, we acknowledge that our observational retrospective study has limitations. The number of patients included was relatively small, there was no control group, data were collected retrospectively, analyses were longitudinal, and the biochemistry samples were uncontrolled.

## 5 CONCLUSION

The conventional approach in the United Kingdom has been to use sodium‐based scavengers to control ammonia in patients with UCDs. However, the arrival of GPB as a glycerol‐based alternative has highlighted the shortcomings of sodium‐based scavengers, particularly their PG content, sodium content, large volume, poor palatability, and relatively short duration of action. Our retrospective observational cohort study of UCD patients initiated on or switched to GPB has demonstrated significant reductions in plasma ammonia, episodes of hyperammonaemia, and hospital admissions. The reasons for these observed findings are multi‐factorial but are likely to include the prolonged action of GPB and its good tolerability, even at higher doses, which facilitates tighter control of ammonia. These findings are consistent with results of other studies.^1^, ^5^, ^6^, ^7^, ^12^, ^13^, ^14^ Overall, we have reduced the exposure of patients to PG and sodium and demonstrated biochemical improvements. These have appeared to translate into positive short‐term clinical outcomes and have the potential to confer longer‐term benefits, as well as improve quality of life.

## CONFLICT OF INTEREST STATEMENT

Mildrid Yeo and Melanie McSweeney have received speaker honorarium from Immedica. The remaining authors declare no conflicts of interest.

## ETHICS STATEMENT

Ethical approval was not required. Informed consent for being included in the study was obtained from the legal representatives of all patients.
